# Application of Optimization Algorithms for Identification of Reference Points in a Monitoring Network

**DOI:** 10.3390/s21051739

**Published:** 2021-03-03

**Authors:** Waldemar Odziemczyk

**Affiliations:** Faculty of Geodesy and Cartography, Warsaw University of Technology, Pl. Politechniki 1, 00-661 Warszawa, Poland; waldemar.odziemczyk@pw.edu.pl

**Keywords:** stability analysis, reference system identification, simulated annealing, metaheuristic method, Monte Carlo method, Hooke-Jeeves algorithm

## Abstract

Geodetic measurements are commonly used in displacement analysis to determine the absolute values of displacements of points of interest. In order to properly determine the displacement values, it is necessary to correctly identify a subgroup of mutually stable points constituting a reference system. The complexity of this task depends on the spatial size of the network, the timespan of measurements and geological conditions affecting the type of changes in the location of points. As a consequence of the abovementioned factors, the task of stable identification in a longer timespan for a subgroup of points may produce equivocal results. In particular, it is likely that alternative subgroups of reference points meeting the mutual stability criteria will be selected, sometimes without common reference points. The proposed method of reference system identification utilises optimisation algorithms. Two such algorithms were tested, i.e., simulated annealing (SA) and Hooke-Jeeves (HJ) method. Two numerical examples were used to test the proposed method. Although in the first example both methods delivered a positive result, the second example showed the superiority of the SA method over the HJ. The proposed method can be considered a tool supporting the person analysing and making calculations in reaching the ultimate decision on reference points.

## 1. Introduction

Determination of point displacements or object deformation can be found in a lot of fields of study, including both engineering (e.g., dam deformations [1,2,3] and natural sciences (deformations related with geological processes [4,5]). Because the effect of these changes may potentially pose a threat to the infrastructure connected with human activity and human life itself, it is often necessary to monitor these changes in order to evaluate the safety levels and predict potential dangers. 

Methods of displacement determination can be divided into geodetic and structural [6,7,8]. Structural methods [9,10] utilise specific equipment such as accelerometers, extensometers, inclinometers and strainmeters [11] and values determined with these devices are relative. Structural methods are often a fundamental feature of automated monitoring systems working in real time.

Geodetic methods utilise a displacement measurement network and provide global information about geometrical features of an object. Geodetic measurements conducted in cycles on the points of the network (in individual epochs) are the source of this information. Displacement networks can be divided into reference or absolute and relative [12]. In absolute networks, it is assumed that at least a part of points is located outside the range of deformations related to the tested object. In that way geodetic methods can provide information about displacements of selected points in the object with reference to an external system.

Although absolute networks contain measurement points located in a way minimising the probability of displacements, with limited knowledge on the actual range of the object’s impact, it cannot be assumed a priori that the points are stable. In absolute networks one of the basic tasks performed during the analysis of measurement results is the verification of reference points stability. It is expected that the procedure will identify a subgroup of mutually nondisplaced reference points, which will be further assumed as stable. On account of the limited accuracy of the measurement, the possibility of gross errors and potentially small values of point displacements, it should be born in mind that an error can occur during the identification of reference points stability. According to Prószyński and Kwaśniak [13], these errors can be classified as follows:

- a type I error (the identified reference base includes only a part of actually stable reference points);
- a type II error (apart from a group of actually stable points, the identified reference base includes the displaced points as well, erroneously identified as stable);
- a combination of type I and type II errors (the identified reference base includes a part of actually stable points only, but with significantly displaced points qualified as stable)

The correct identification of a reference system is key from the point of view of further determination of displacements of the tested object. Qualifying the displaced points to the reference system (a type II error) lead to determining a false displacement of the object, which in turn might result in erroneous conclusions about the safety of the object [14]. 

Numerous identification methods of a reference system are known. Identification can be conducted as part of a process of determining the displacements or as a separate process preceding the calculations of displacements [13]. In the first case, it is usually an iteration procedure leading to the identification of an ultimate group of stable points. The completion of this process is simultaneously the identification of both the reference system and the determination of ultimate displacement values of stable points. It is exemplified in the procedures described in [15,16,17].

In the second case, the coordinates of potential reference points determined independently for two (or more) epochs and their accuracy characteristics are available. The data usually come from an independent preliminary adjustment of individual epochs. In this case, the identification of a reference system involves an analysis of geometrical features of a group of points of a potential reference system and can have the form of a searching transformation method, for instance. Algorithm of numerical control of object adherence [18], Global Congruency Test [19,20,21,22] or Iterative Weighted Similarity Transformation (IWST) [23] are all examples of this method. The paper by Mrówczyńska [24] includes an example of identification of a reference system for a levelling network. 

Independently of the method employed, the identification of a group of stable reference points can be a difficult task and its results can be equivocal. This may occur with vast objects, particularly in geologically differentiated area in which the reference network is stabilised [25,26]. Additionally, the variable direction of causes of displacements may increase the risk of difficulties. All of the above may lead to a situation in which more than one group of mutually stable reference points is identified, but the group is mutually unstable towards other groups [13]. It can be easily shown (see [28]) that the Global Congruency Test does not perform well in such case. In the best case it finds one of the groups of stable reference points. It is of utmost importance to be aware of the fact that different groups of mutually stable reference points exist. As the problem has significant practical implications, several stability identification methods were developed. Apart from the above quoted article of Wujanz et al. [26], which describes the identification of stable areas in a laser scanning point cloud, works of Neitzel [27,28] and the publication of Lehmann and Lösler [29] should also be mentioned. The methods described in those works are based on stability testing of the selected subgroup of reference points. In both cases a combinatorial procedure is used to select individual subgroups. Such an approach guarantees that even small subgroups can be detected but it has also some flaws. At first, complete analysis of the large network implies computational costs which grow exponentially according to the number of points. It should also be noted, that Neitzel’s method uses a subset of observations for the analysis of the point congruency, which fastens the whole procedure but weakens the accuracy of point positions.

The identified groups of reference points facilitate the determination of parameters of transformation between the systems of coordinates for both epochs. The number of these parameters depends on the size of the network and, if the scale is stable for both systems, amounts to 1—for a vertical network, 3—for a flat network and 6—for a spatial network. Transformation parameters create a certain one-, three- or six-dimensional space of solutions. Each point of this space corresponds to a set of transformation parameters and consequently to the set of residuals on all reference points.

A new alternative method of constant point identification is based on such an approach. The main idea of this method stems from the fact that the identification of the reference system may be seen as the search for a point in the space of transformation parameters. The key for the search is the properly defined objective function utilizing transformation residuals on reference points and assessing the analysed point of the transformation parameters space. This approach is somehow analogous to the Hough transform [30] utilised to identify geometrical objects in a point cloud data in computer vision.

The proposed method utilises optimisation algorithms. The computational cost is usually high in the case of such algorithms but contrary to the combinatorial methods, it doesn’t grow so rapidly with the number of points. The computational cost in the proposed method is proportional to the number of network points while in case of combinatorial methods it grows exponentially. For example, assuming a minimal number of 2 stable points, we obtain the following number of combinations to be tested: 16,365 for 14 points [29], 1,048,551 for 20 points and 1,073,741,786 for 30 points.

On the other hand, the coordinates of the reference points are the result of preliminary adjustments of the whole set of observations for each epoch, which improves the internal accuracy of the network.

Due to the unique nature of the task, two algorithms were selected from among numerous others: the metaheuristic algorithm of simulated annealing (SA) and the Hooke-Jeeves algorithm (HJ). The first of those algorithms comes from thermodynamics and reflects the process of solidification of a liquid metal into a crystalline solid. It is considered as calculation cost consuming. It was chosen due to its ability to get the global minimum of the objective function avoiding the existing local minima. Section 2.1 contains a detailed description of the SA algorithm. The second algorithm has a totally different—deterministic—character. For the same starting point in the search space we always obtain the same final result—the local or global minimum of the objective function. The HJ algorithm is relatively fast but it is prone to local minima which will be found as the solutions. Detailed description of the HJ algorithm is presented in Section 2.2. If more than one group of constant reference points exists, we can expect more than one minimum in the search space. To identify all of them a hybrid solution must be used in which the starting point for the HJ procedure is selected randomly. The use of both algorithms is described in detail in Section 2.4.

For each of these algorithms an objective function had to be formulated, which reflected the quality of coordinates fitting into both analysed epochs. An original procedure was suggested for this purpose.

The proposed method can be applied for levelling (1D), horizontal (2D) or spatial (3D) networks. In case of 1D network the problem is quite simple and is limited to the search of optimal height shift between the epoch height systems to reach desirable fitting on the selected reference points. Except for LIDAR method, 3D monitoring networks are rarely used due to problems with integration of various measuring techniques. The identification of stable regions in LIDAR point clouds needs special approach considering a large number of points and the fact that different points are registered in particular epochs [26].

For that reason 2D test objects are selected to illustrate the performance of the proposed procedure. Two simulated two-epoch tests of an object consisting of many subgroups of stable points were used. The results of the tests are presented in Section 3. The obtained results are discussed in Section 4.

## 2. Materials and Methods

### 2.1. Simulated Annealing

Common use of computer calculation methods and the related decrease in the cost of computational operations caused reconsideration of the way we look at estimation tasks and algorithms used for these purposes. This new approach resulted in new methods from the Monte Carlo or Metaheuristics Family, in which numerous repetitions of a computational sequence are required to obtain the results.

Simulated annealing, also known as Monte Carlo annealing, probabilistic hill climbing, or stochastic relaxation belongs to metaheuristic methods. The idea behind SA comes from thermodynamics and reflects the process of solidification of a liquid metal into a crystalline solid. In this process the molecule mobility decreases with the decrease in temperature, and if the cooling rate is sufficiently slow, the molecules can achieve the state of mutual order corresponding to the lowest energy state (e.g., create crystal lattice).

**Figure 1:**
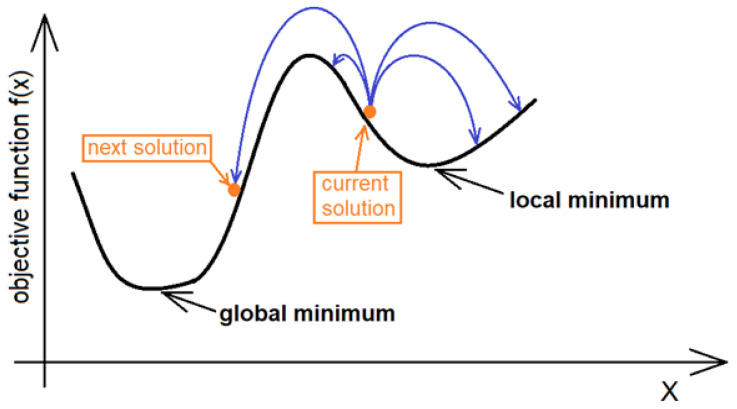
Idea of solution seeking for one-dimensional objective function [34].

Since the SA algorithm was first published by Metropolis et al. [31] and developed by Kirkpatrick et al. [32], it was used to solve a wide variety of problems. The most useful characteristic of annealing process reflected in the algorithm structure appeared to be the ability to avoid local minima corresponding to the pseudo-crystalline state with the energy level higher than minimal. Figure 1 illustrates the idea of a search for solution for one-dimensional objective function. More information on the SA algorithm can be found in [33].

The properties of the SA algorithm, especially its ability to solve complex optimization problems with local minima, facilitated its use in areas sometimes really far from thermodynamics. Solving the well-known travelling salesman problem is frequently used as an example [35,36]. 

Some metaheuristic methods have also been used to solve some optimization problems in geodesy and surveying techniques, e.g., [37,38,39], but applications of SA in this field are rather limited. They include research on geodetic network design [40,41], the adjustment of geodetic measurements [42,43], LIDAR survey design [44] or coordinate transformation [34]. Deformation measurements which are the subject of this work can be included in the same area.

SA is an iterative algorithm, in which the continuous change in the temperature of a cooling liquid is replaced by incremented changes introduced in subsequent iterations. Its use for solving estimation tasks requires a definition of several essential elements:

1. An objective function corresponding to the molecular energy level during the annealing process that will be minimised;
2. A cooling scheme, comprised of an assumed initial temperature and dependency defining temperature drop after each iteration;
3. A molecule movement model corresponding to the actual temperature;
4. Termination criteria for the iterative process. The condition can be formulated based on:
  - the final temperature (minimal)
  - the acceptable value of the objective function
  - the range of the molecule movement, which usually corresponds to the estimated model parameter changes defining the objective function

**Figure 2:**
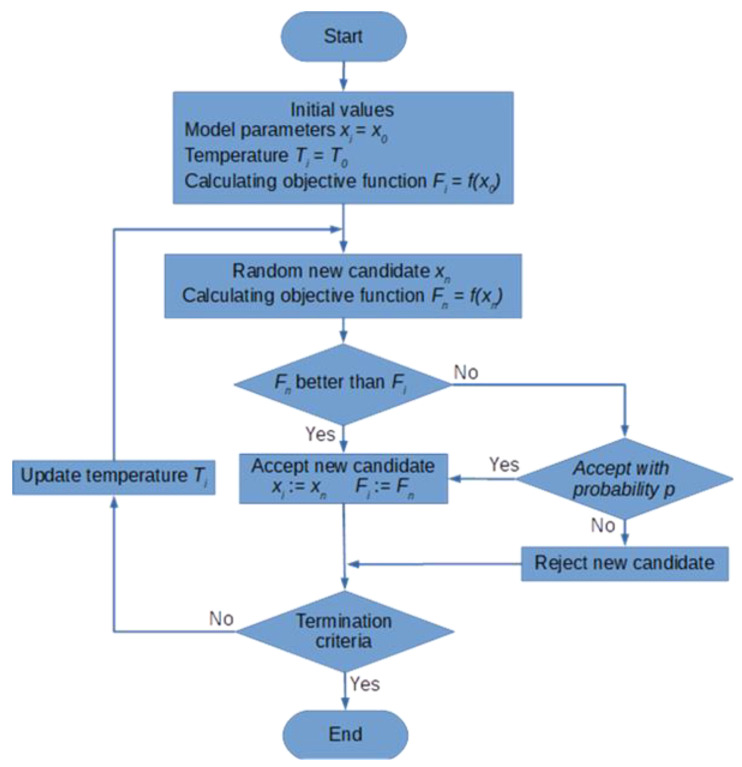
Flowchart of the simulated annealing method [34].

A detailed flowchart of the algorithm is shown in Figure 2. By defining: *x_i_*—vector of the current model parameters in the *i*^th^ iteration of the estimation task, *f*(*x_i_*)—objective function defined for the current parameters, *Δx_i_*—change in parameter vector in the *i*^th^ iteration, the elements of the simulated annealing algorithm can be presented as follows:

#### 2.1.1. Objective Function

The objective of the algorithm is to find the global minimum. Therefore, the objective function *f*(*x_i_*) must provide an answer whether the current solution (*x_i_* vector) is better than the previous one and, thereby, if it is closer to the final solution which corresponds to the minimum of the objective function. The definition of the objective function will be discussed in a separate section.

#### 2.1.2. Cooling Scheme

The temperature as a parameter of the SA algorithm stems from physical analogies. In optimization tasks, the value which allows to easily control the solution-seeking process is assumed as the temperature equivalent.

The core of the cooling scheme lies in the function of the temperature change. In such a situation, it is more important to define the function of the temperature change. Various schemata are used in the SA algorithms, but they always take the following form:

*T* = *f*(*T*_0_, *i*), (1)

where *T*_0_ is the initial temperature and *i* is the iteration number. As the temperature is tightly connected with the iteration number, in some cases it is possible to replace the temperature parameter with the iteration number.

#### 2.1.3. Molecule Movement Model

The movement model determines the way the new solution is obtained. It is generally defined by two elements. The first of them is the way the next (subsequent) solution is generated. It is described by the Equation (2).

*X*_*i* + 1_ = *x_i_* + Δ*x_i_*, (2)

Δ*x_i_* vector is randomly generated and usually a normal distribution N(0, σ) is used. The current standard deviation of this distribution *σ_i_* is the function of the initial value *σ*_0_ and temperature (3):

*σ*(*t*) = *σ*_0_*β^t^* (3)

or

*σ_i_* = *σ*_0_*β^i^* (4)

*β* coefficient corresponds to the cooling speed and assumes values in the range (0,1) and *t* defines the time which—in practical solutions—can be replaced by the iteration number *i*. 

The second key element of the molecule movement model is the criterion for accepting a new solution. The decision-making process of accepting the new solution as the current one (potentially the best) consists of three phases. Firstly, it is checked whether the obtained solution (*x_i_* + Δ*x_i_*) belongs to the task field—in other words, whether it fulfils the formal requirements of the task being solved. Secondly, the value of the objective function is analysed:

(5) $$x_{i+1}=\left\{\begin{array}{cc} x_{i}+\Delta x_{i} & if\;f\left(x_{i}+\Delta x_{i}\right)<f\left(x_{i}\right)\\ x_{i}+\Delta x_{i} & if\;f\left(x_{i}+\Delta x_{i}\right)\geq f\left(x_{i}\right)\;\mathrm{with}\;\mathrm{probability}\;p\;\\ x_{i} & \mathrm{otherwaise} \end{array}\right.$$

If the obtained value of the objective function is lower than the current value, the new solution (*x_i_* = *x_i_* + Δ*x_i_*) is accepted unconditionally. Otherwise, even though it corresponds to a worse solution from the objective function’s perspective, the obtained vector is accepted with a determined probability *p*. The most advanced method—based in the thermodynamic origins of the procedure—is the application of Boltzman distribution. The value of *p* is then defined by the Equation (6).

(6) $$\;p=e^{-\frac{f\left(x_{i+1}\right)-f\left(x_{i}\right)}{T_{i}}}$$

This method gives relatively high *p* values, and, on the one hand, it provides a higher guarantee of finding the global minimum. On the other hand, frequent acceptance of a worse solution considerably slows down the iteration process. A simpler solution would be to assume a constant value for *p*—most commonly ranging from 0.001 to 0.2 (like in [41]). In the simplest tasks with uncomplicated spatial distribution of the objective function this element of the algorithm can be omitted, which corresponds to the original version proposed by Metropolis team [31].

#### 2.1.4. Termination Criteria for the Iterative Process

Termination of the solution seeking process may be linked to acquiring a specific temperature value, which corresponds to a certain number of conducted iterations. Another option may be reaching a satisfactory value for the objective function. In both cases, it is essential to determine the critical value. It depends on the required accuracy of the solution. In special cases, the SA method may be used to determine an approximate solution that will subsequently allow for the application of other methods utilising, e.g., linearization of the functional dependencies describing the given task. In this case a significantly lower number of iterations is required in order to obtain a satisfactory result.

### 2.2. The Hooke-Jeeves Method

The Hooke-Jeeves (HJ) local search algorithm method was proposed by Hooke and Jeeves in 1961 [45]. Because it is a pattern search method, it can be used when the objective function is irregular.

The point of departure for the Hooke-Jeeves method is to define the following parameters:

***d***—the orthogonal n base for linearly independent orthogonal vectors,
*τ*—the initial length of the searching step dependent on the area of searching and the distribution of the objective function,
*γ*—the ratio of decreasing the searching step,
*τ_end_*—the minimal length of the step which is the criterion of the end of the searching process,
*x*0—the starting point of the procedure.

Each iteration in the HJ method consists of two moves:

- **the exploratory move**, in which the distribution of value of the objective function is tested within a small selected area of the base point, utilising trial steps along all directions of the orthogonal base ***d***;
- **the pattern move** involves moving in a strictly determined manner to the next base point in which another exploratory move is considered, but only on condition that at least one of the trial steps taken was successful.

A step is successful if it leads to the decrease in the value of the objective function. If none of the steps were successful, you return to the previous base point and the search cycle starts again with a decreased length of step *τ*.

**Figure 3:**
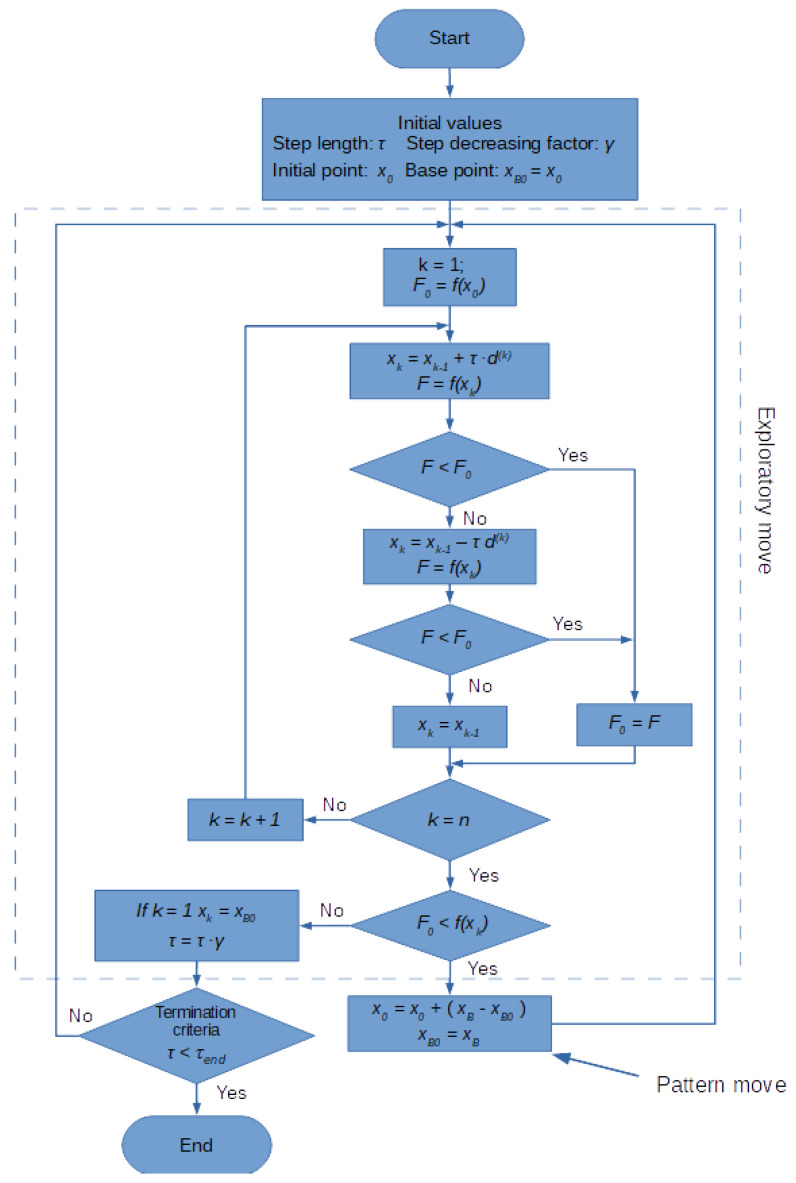
Flowchart of the Hooke-Jeeves method.

The algorithm ends its work as soon as the ratio of the step *τ* achieves the assumed final value *τ_end_*. The HJ algorithm is shown in detail in Figure 3. *f*(*x*) stands for objective function.

Unlike the SA method, the HJ method is totally deterministic. The same solution will always be obtained for a given starting point and the same search parameters.

The HJ method is simple and relatively fast-converging, which, in combination with no need to calculate the gradient of the objective function, makes it attractive if the objective function has no analytical form and is obtained based on the empirical data. The practical applications of the HJ method include different fields of widely understood engineering [46,47,48,49,50]. The method is not very popular in geodesy and measurement data processing. Work [51] is the exception in this area.

### 2.3. The Objective Function for Identifying the Reference Base

The objective function plays a key part in optimisation tasks. It has to be formulated in such a way that one numerical value determines the level of the achieved objective, i.e., a set of parameters corresponding with the searched optimum. It is usually assumed that the objective function amounts to a minimal value in a point being a solution to the optimisation task. Moreover, it is desirable that the distribution of the objective function in the space of parameters facilitates the choice of an optimal route to the objective. 

In accordance with the assumptions made in this work, the search space for the solutions is the space of transformation parameters. As it was mentioned before, the space can be one-, three- or six-dimensional. The following Equation (7) describes the transformation of coordinates:

*b_i_* = *Ra_i_* + *t*. (7)

In the case of horizontal network its elements can be defined as follows:

*a_i_* = [*x_i_*, *y_i_*]*^T^*—position vector for the point in the starting system of coordinates, *b_i_* = [*X_i_*, *Y_i_*]—position vector for the point in the final system, *t* = [Δ*X*, Δ*Y*]*^T^*—translation vector. *R* is a rotation matrix with dimensions 2 × 2. Its four elements are the functions of one rotation angle *α* (8)

(8) $$R=\left[\begin{array}{cc} \cos\left(\alpha \right) & -\sin\left(\alpha \right)\\ \sin\left(\alpha \right) & \cos\left(\alpha \right) \end{array}\right].$$

Assuming that vector *a_i_* in Equation (7) corresponds with the coordinates of point *i* for the first epoch, and vector *c_i_* with coordinates for the same point for the second epoch, residuum *r_i_* (9) can be determined for each point

*r_i_* = *c_i_* − *b_i_*. (9)

Modules of residual vectors |*r_i_*| will provide the basis for defining the objective function. 

As far as the identification of the reference system is concerned, formulating the objective function is quite a complex issue. This is because in this case the aim is to find a certain and sufficiently numerous subgroup of reference points which will be mutually consistent with the internal geometric features in both measurement epochs.

The term constancy must be considered in relation to the set of transformation parameters (Δ*X*, Δ*Y*, *α*) which also determine a point in search space. Each such set allows us to make coordinate transformation (7) and calculate a set of residuals for individual points (9).

Consistency occurs when the inequality (10) is satisfied for the current point or, in other words, when the module of its residual does not exceed the assumed critical value. In the simplest variant, this critical value *ε_i_* is a constant value. However, if covariance matrices of coordinates of points for individual epochs are used, this parameter will take into account positional accuracies of the considered point in both epochs (11)

|*r_i_*| < *ε_i_*, (10)

(11) $$\;\varepsilon_{i}=2\sqrt{\sigma_{Pi\left(1\right)}^{2}+\sigma_{Pi\left(2\right)}^{2}}\;.$$

where *σ_Pi_*_(1)_ and *σ_Pi_*_(2)_ are point position errors in epochs 1 and 2, respectively.

(12) $$\;\sigma_{Pi\left(1\right)}=\sqrt{Q_{ix\left(1\right)}+Q_{iy\left(1\right)}}\;\;\;\;\;\;\sigma_{Pi\left(2\right)}=\sqrt{Q_{ix\left(2\right)}+Q_{iy\left(2\right)}}$$

*Q_ix_*_(*n*)_, *Q_iy_*_(*n*)_—the element of covariance matrix for epoch n corresponding with *x* and *y* coordinates of point *i*.

To simplify things, a group of points meeting the condition (10) will be called consistent points. If all the points meet condition (10), they can be considered stable points of the displacements monitoring network in two epochs. It is the most desirable case but in real life we have to assume that only some of the points can maintain constant positions. In such a case obtaining consistency for a satisfactory numerous subset of points means that the current coordinate transformation parameters describe the transformation of the network reference system.

Considering the way the optimization methods find a solution, the following postulates can be formulated:

- The objective function should generate considerably stronger signal (value decrease) for those points in the space of transformation parameters for which the group of consistent points is obtained.
- It should be insensitive to the points not belonging to the identified group of consistent points.
- It should “promote” a larger size of the group of consistent points (the value of the objective function should be lower for a larger subgroup of consistent points than for a smaller one).

**Figure 4:**
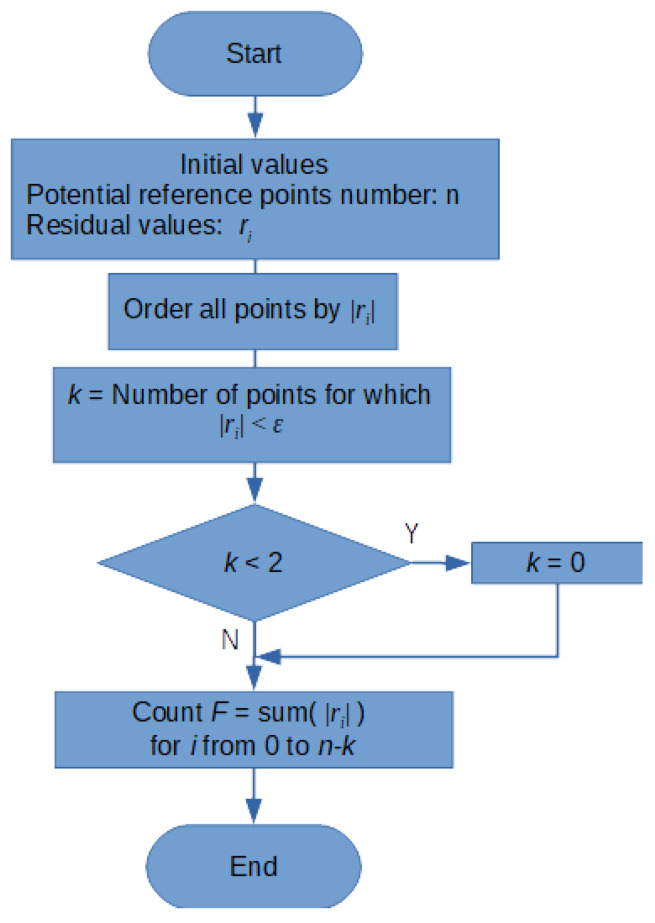
Flowchart of the objective function calculating algorithm.

The special algorithm for calculating the objective function was proposed to fulfil the abovementioned requirements. Its flowchart is depicted in Figure 4. The algorithm is based on the sum of absolute values of residual vectors calculated for all (n) potential reference points. If a certain k-point subgroup is consistent, the points with the largest residues are rejected from the sum. The number of rejected points is equivalent to the number of points with residual vectors fulfilling the consistence criterion. 2 is the minimum group of consistent points. Consistency for a single point does not mean reinforcement (the point with the largest residues is not rejected). It is worth noting that if all reference points (k = n) are consistent, the objective function will equal 0.

The finally calculated F value is the objective function value for transformation parameters set used to calculate residuals according to the Equation (9). This value is then used in both optimization algorithms as *f*(*x*) function.

### 2.4. Numerical Implementation of Identification Algorithms for Reference Basis

The transformation of spatial coordinates is described by Equation (7). If vector *a_i_* is a position vector of a point (referring from the origin of the coordinates system), a translation vector *t* means the translation of the origin of the system of initial coordinates and therefore the position of this point in the final system. This situation is unfavourable since the values of the components of the translation vector *t* may significantly deviate from the real movements of the points in the object. This might happen especially if these points are located considerably far from the origin of the system of coordinates, and the rotation angle *α* has significant values. Therefore, the concept of the base point of transformation is often utilised during the practical implementation

*b_i_* = *R*(*a_i_* − *a*_0_) + *b*_0_ + *u*. (13)

Here, vectors *a*_0_ and *b*_0_ are position vectors of the base point for epochs 1 and 2, respectively. Vector *u* = [ *dx*, *dy* ] is the residual translation vector. It would be most beneficial to adopt the centre of mass of the group of potential reference points as the base point. 

Utilising the optimisation algorithms described in previous sections to identify the reference points requires specifying the key parameters for their operations and connecting these parameters with the parameters of the practically implemented task. 

As mentioned before, the area of search will be a three-dimensional space determined by transformation parameters—in this case, components of vector *u* and rotation angle *α*. 

The other parameters will be defined depending on the used algorithm.

#### 2.4.1. The Simulated Annealing Method 

The search range in this method should be specified as referring to component search ranges—*δx*, *δy*, *δα.* The range facilitates the limitation of the number of analysed solutions (points of search area). What is more, it is strictly connected with the controlling parameter—an equivalent of temperature. Owing to the transformation model with a base point (13), the range of the first two parameters can be determined based on the maximum possible displacements of reference points *δx*_0_ = *δy*_0_ = *δp_max_*. In the torsion angle, the following dependency can be assumed:

*δα*_0_ = arctan(2*δp_max_*/*d_max_*), (14)

where *d_max_* is the maximum distance between the potential reference points. The controlling parameter is an independent coefficient *t*. Its initial value amounts to *t*_0_ = 1. The value of *t* in w the—*i*^th^ iteration amounts to:

*t_i_* = *t_i_*_− 1_*β* = *β^i^*^− 1^, (15)

where *β* is the cooling coefficient. 

The range of search in the *i*^th^ iteration amounts to:

*δx_i_* = *δx*_0_* t_i_*, *δy_i_* = *δy*_0_*t_i_* and *δα_i_* = *δα*_0_*t_i_* (16)

respectively.

Values *δx_i_* and *δy_i_* will be used to formulate the criterion of the end of the iteration process. 

In the task described, adopting an appropriate value of the cooling coefficient *β* is of crucial importance. Adopting a value too close to unity will result in the procedure returning a global minimum mainly, omitting local minima. In the case discussed, the dominant subgroup of reference points with the lowest value of the objective function is equivalent to the global minimum. As the objective set at the beginning is the identification of all possible subgroups, it is advisable to apply a bit faster pace of cooling, which will enable us to detect the local minima as well. The value of the cooling coefficient is best determined with an empirical method.

Inequality *δx_i_* (*δy_i_*) ≤ 0.001 m was adopted as the final criterion of the iteration process. It should be estimated that the value adopted in the final criterion does not translate directly into the accuracy of the final result. 

#### 2.4.2. The Hooke-Jeeves Method

As it was mentioned before, the HJ method algorithm is of totally deterministic nature, which means there is a strong connection between the origin and the obtained solution. To meet the initial assumption, i.e., to identify various local minima of the objective function in the search space, random selection of the starting point was used. The components of the position vector of this point in the search space are as follows:

*dx*_0_ = *rnd*(*δx*_0_), *dy*_0_ = *rnd*(*δx*_0_), *α*_0_ = *rnd*(*δα*_0_), (17)

where *rnd*() is a random function with a uniform distribution.

Considering the above assumptions, one may expect that there is a nonzero group of starting points for each local minimum, leading to the minimum in question. 

As the HJ algorithm approaches equally all components of the search space, it is indispensable to coordinate units for these components. While the first two components refer to the flat coordinates and are equal by nature, the third coordinate corresponding to the rotation angle is completely different. In particular, it is necessary to specify the working unit for the rotation angle corresponding with the coordinate unit. The value of this unit is not constant and depends on the size of the network. Its value will be calculated using the Equation (18).

1u*α* = 1 m/*d_max_* (18)

Thanks to the harmonisation of units, it is possible to search the solutions space using the same value of *τ* jump for all the components. 

Moreover, the following parameters should be adopted for the HJ method:

*τ*_0_—initial length of the search step
*γ*—coefficient of the search step decrease
*τ_end_*—criterion of the end of the search process (minimum step size)

The following values were adopted for the task in question:

*γ* = 0.8
*τ_end_* = 0.0001 m(u*α*)

where u*α* is a working unit of the angle adopted for the coordination of units in all three components (18).

The described algorithms were implemented in author-created software. Borland Delphi programming environment and Object Pascal programming language were used. Such an approach made it possible to control all the important parameters and have an insight into the details of the identification process.

## 3. Results

To test the validity of assumptions, a series of analyses was conducted on two simulated testing examples. To check the effectiveness of the identification of subgroups of mutually stable reference points in all points of each object, subgroups were isolated for which different displacements were simulated.

### 3.1. Test Example 1

**Figure 5:**
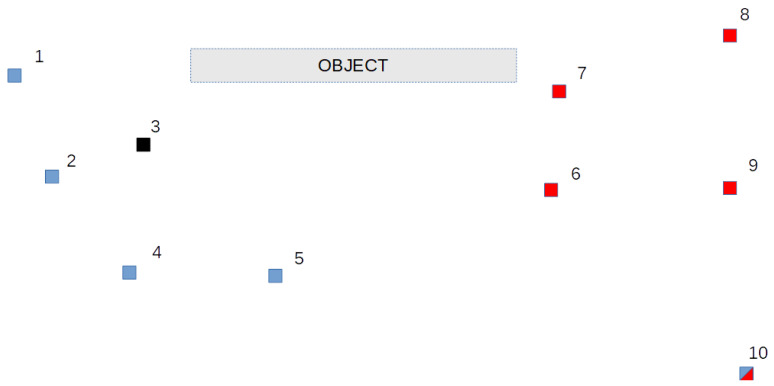
Test network 1 points placement.

The first test network consists of ten points constituting a reference network to test displacements of the imaginary object. The placement of the points in the network is depicted in Figure 5.

Two subgroups of mutually stable points were simulated for this network. The first one includes points 1, 2, 4, 5 and 10, the second—6, 7, 8, 9 and 10. Point 10 is a common point for both groups, whereas point 3 is a nonstable reference point for each of the mentioned subgroups. The resulting coordinates for epoch 2 were disturbed by simulated errors with a normal distribution and standard deviation *σ_p_* = ±2 mm. Such a value is an equivalent of disturbance in both epochs with errors of ±1.4mm standard deviation.

The coordinates of points of the test object for both epochs are presented in Table 1.

Before the test of optimization algorithms a commonly known congruency test will be conducted. It consists of a series of three-parameter 2D transformations. The standard deviation of the standardized residuals is compared with its critical value and used as the congruency index of the current subset of points. If the test fails (standard deviation is larger than its critical value), one point with maximal residuals is excluded from the current subset. The critical value is calculated using chi-square test assuming a significance level *α* = 0.05 and a degree of freedom *f* = 2*n* − 3 (*n—*number of points in the current subgroup). Table 2 shows the course of the procedure.

For omitted subgroup of points (6, 7, 8, 9, 10) the corresponding values are: *σ_xy_* = 1.06 with *σ_xy max_* = 1.42.

The same data were used in testing the effectiveness of the two optimization methods described in Section 2. The test involved *n* = 1000 trials. The identification results are juxtaposed in the tables. The following values of simulated annealing parameters were adopted for the test object 1:

*δx*_0_ = *δy*_0_ = 0.05 m
*δα*_0_ = 0.002 gon
*β* = 0.997

To determine *δα*_0_, formula (14) was used, assuming *d_max_* = 3000 m.

The results obtained with the SA method are presented in Table 3. Apart from the identified groups of stable points and the frequency of their identification, the Table includes the evaluation of the mutual consistency of points placement as standard deviation (*σ_o_*) of coordinates residuals obtained in a classic, three-parameter 2D transformation for the points of a given group.

The approximate computation time of 9 s was necessary for all 1000 trials. Repeating the test shows that the percentage of hits is quite stable. The instability does not exceed 1%.

1u*α* = 0.00033 rad ≈ 0.02 gon and *τ*_0_ = 0.05 m(u*α*) was taken for the Hooke-Jeeves method. Test results for the HJ method are presented in Table 4.

The approximate computation time for n = 1000 trials is about 12 s. The stability of hits for multiple call of the procedure is about 2% for two main solutions, while the minor solutions are unstable and change as far as the subgroup and the number of hits.

### 3.2. Test Example 2

**Figure 6:**
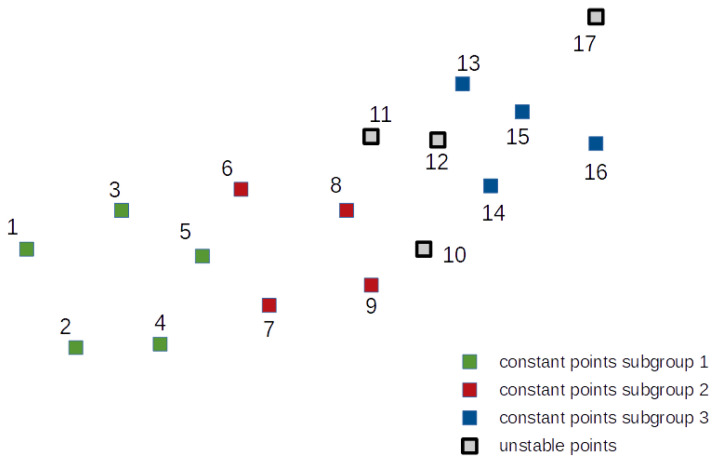
Test network 2 points placement.

The second test object is slightly more complex. It consists of 17 points which create three subgroups of mutually stable points. Four points were simulated as unstable and do not belong to any subgroup. The placement of the test object points with their belonging to the individual subgroups is shown in Figure 6.

Three subgroups of mutually stable points were simulated for this network. They are shown in Figure 6 and consist of points 1 to 5 (subgroup 1), points 6 to 9 (subgroup 2) and points 13 to 16 (subgroup 3). The rest of the points are nonstable. The coordinates for epoch 2 were disturbed by simulated errors with a normal distribution and standard deviation ±5 mm.

The coordinates of points of the test object 2 for both epochs are presented in Table 5.

The following values of simulated annealing parameters were adopted for the test:

*δx*_0_ = *δy*_0_ = 0.1 m
*δα*_0_ = 0.01 gon
*β* = 0.999

To determine *δα*_0_, dependency (14) was used, assuming *d_max_* = 1000 m.

1u*α* = 0.001 rad ≈ 0.06 gon and *τ*_0_ = 0.1 m(u*α*) was taken for the Hooke-Jeeves method.

Similar to the test example 1, the test involved n = 1000 trials. 

The results obtained with both methods are presented in Table 6 (SA) and Table 7 (HJ).

The approximate computation time for n = 1000 trials is about 100 s. The multiple call of the procedure shows that the stability of hits is about 2% for each of the identified subgroups.

The computation time for n = 1000 trials is about 15 s. The multiple call of the procedure shows that the stability of hits is about 3–4% for most of the solutions. That means that there is quite a large group of solutions which appears randomly in particular trials.

## 4. Discussion

The test objects shown in the previous section differ in total number of points and number of subgroups of constant points. As formulated in Section 2.3, the main purpose of the objective function definition was to detect points in the search space which correspond to the possibly numerous subgroups of consistent points. Such a situation can be found in the test object 1. In the test object 2 constant point subgroups consist of a smaller number of points, while the whole number of points is larger. The difference affects significantly the identification procedure performance.

### 4.1. Test Object 1

This test object consisted of two, equally numerous subgroups of constant points. Each subgroup accounted for a half of all points with one point which is common for both groups.

An experiment with a classical approach when the following points are excluded from the reference base until standard deviation test is passed proved that only one subgroup of stable points can be detected at the end of the process. The choice of the group which will be finally chosen depends of various factors and is random to a large extent.

As can be seen in the Table 3, the simulated annealing method only provided solutions corresponding with the assumed groups of stable points. Both groups were indicated almost equally frequently. It is also worth noting that the dominant subgroup has a better index of internal consistency. However, it should be borne in mind that the objective function constituting the basis for the identification of solutions does not use the sum of squares of adjustment residuals used for calculating σ_0_.

The HJ method (Table 4) also led to the identification of both assumed groups of stable points. However, unlike the SA method, it resulted in erroneous solutions in ten cases. The likely reason behind this behaviour of the algorithm is additional, subtle local minima of objective functions. The minima do not pose a problem in the SA method dedicated exactly to solving such tasks.

It is worth noting that erroneous solutions constitute merely 1% of cases and can be easily eliminated in the course of statistical analysis of the identification results. The HJ method, unlike the SA method, identified more frequently the first group of points. It must be noted that in the HJ method the difference is quite significant—62.3% as compared with 36.5%. For the SA method, those values amounted to 55.4% and 44.6%, in favour of the second group.

**Figure 7:**
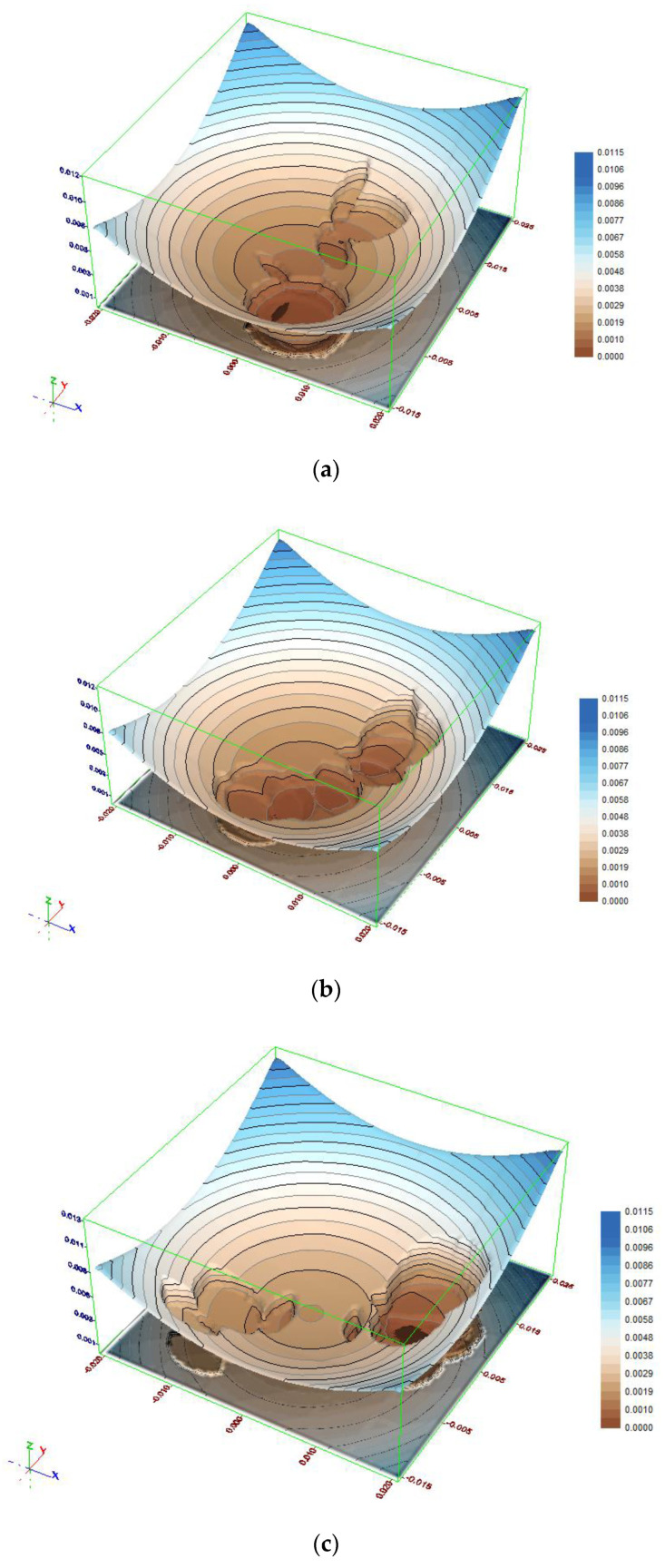
Distribution of the objective function for test object 1 (**a**) *δα* = 0.0 gon; (**b**) *δα* = 0.00035 gon; (**c**) *δα* = 0.0007 gon.

To better understand the obtained results an analysis of the objective function will be helpful. The objective function distribution for the test object 1 data is three-dimensional and per se it is difficult to depict it in a 2D figure. Figure 7a–c depict the value distribution for three cross-sections of the search space. These are *δα* = 0.0 gon (a), *δα* = 0.00035 gon (b) and *δα* = 0.0007 gon (c), respectively.

In the figures one can notice distinct, irregular concavities in the regular objective function distribution. These concavities correspond with groups of fixed points and result from rejecting the outlying points, which leads to a step decrease of the objective function.

The places in the objective function distribution corresponding to the constant points subgroups are clearly visible (especially in Figure 7a,c). Such a situation allowed both methods to achieve success. The most important circumstance was the fact that both subgroups included nearly half of the total number of points, and as a consequence the objective function was in a very small part affected by the points outside the considered group.

### 4.2. Test Object 2

**Figure 8:**
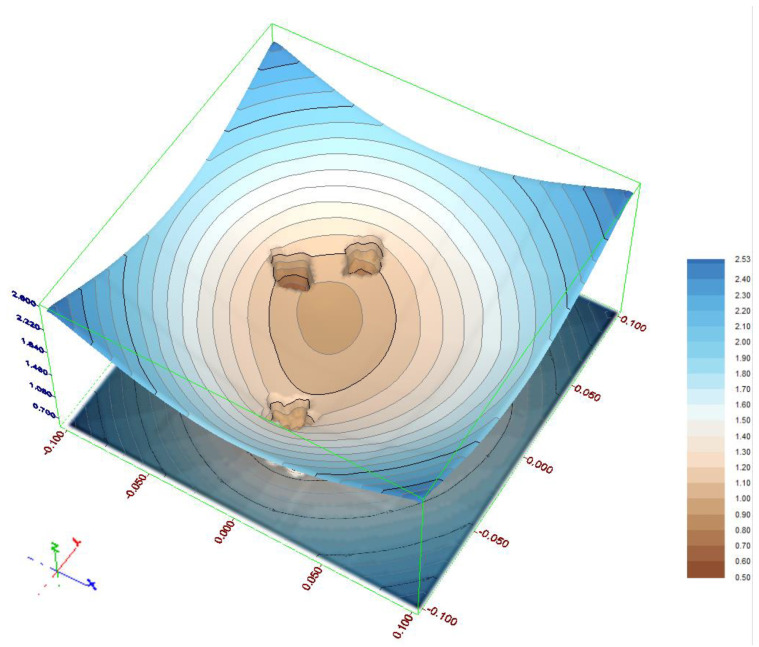
Distribution of the objective function for test object 2 for *δα* = −0.0025 gon.

The second test object consists of three subgroups of constant points and a group of points which do not belong to any group. The most important difference arises from the fact that even in the case of the most numerous subgroup the objective function will be strongly affected by the rest of the points. Such a situation had an influence on the identification results. Because of the larger displacements assumed, the *δx*_0_ and *δy*_0_ parameters needed to be enlarged to 0.1 m. Due to more complex objective function distribution (Figure 8) the cooling factor of the SA algorithm was set to 0.999.

As can be seen in Table 6, the SA method delivered only three different combinations of points. All of them are assumed constant point subgroups. Unlike in the Test object 1 the probability of detection is considerably diverse. It appeared that the subgroup consisting of points 13–16 was most frequently detected (75.1%). The subgroup created by points 6–9 is detected with probability 20.8% and the most numerous group made by points 1–5 was detected with least probability (4.1%).

Analysing the results of the HJ method we can see that it failed in a large number of trials. Although the largest assumed subgroup (or the majority of its subsets) are a dominating part of the solutions, a large number of erroneous results leads to the conclusion that the HJ method is not a proper solution for the considered task with this kind of the objective function. This is mainly due to the irregular objective function distribution.

The result of the SA method can be considered as a success but it also showed some limitations of the proposed method using the objective function described in Section 2.3. As it was assumed, the objective function is oriented at finding rather large subgroups of points. It is common in the real objects monitoring when reference network is properly designed and such groups ensure that the deformation of the object will be accurate and reliable. The situation where only a minor subgroup of potential reference points keeps its stability can appear in the case of a catastrophe which causes a large extent of changes. In such a case combinatorial methods described in [27,28,29] will be a better solution.

The existence of many subgroups of constant points in the HJ procedure results suggests that the second step analysis is necessary. Its main task is to unite the detected subgroups. The subgroup being the subset of the more numerous subgroup should be included in the larger subgroup. This will reduce the number of subgroups and make the results overview clearer.

Another aspect that must be explained is the computational cost reflected by the computation time. For the test object 1 both methods needed similar time for the solution. (9 s—SA, 12 s—HJ). A completely different situation appeared in the case of test object 2. While HJ obtained the solution in a slightly longer time than for the object 1 (15 s.) The time necessary for SA was over 10 times longer (100 s). The differences arise from three elements: number of points, the extent of the search space fragment being examined and the internal parameters of the particular algorithms.

The moderate growth of the HJ algorithm operating time allows us to conclude that the number of points as well as the search space extent did not have the significant influence on the large growth of the SA algorithm operating time. The cooling factor appeared to be crucial. The seemingly small difference in the value of the cooling factor translated into large growth of iteration number (1303 for the test object 1 and 4603 for the test object 2). Such increase in the computational cost appeared to be necessary to obtain the exact solution for the test object 2.

Although numerical tests showed that the proposed method based on optimization algorithms and using the proposed objective function can be applied for the identification of constant points in a monitoring network, further research seems to be necessary. The most promising direction of the research is to formulate a more efficient objective function which will be less sensitive to the movements of the unstable points and which will not generate the local minima corresponding to the subsets of the larger constant points subgroups.

## 5. Conclusions

The proposed procedure of identification of the reference system based on the search of the space of transformation parameters was intended not only to identify the group of mutually stable reference points, but also to detect the potential alternative solutions. The procedure was based on the special form of objective function and two selected optimisation methods. 

The conducted analyses and two numerical experiments proved the usefulness of the optimisation procedure. The experiments showed that the Hooke-Jeeves algorithm can be used only in relatively simple cases, when a great part of reference points keep the stability. The simulated annealing algorithm performs well in a wider range of situations. It is able to detect even minority subgroups of reference points and is less likely to produce erroneous result. Another advantage of the SA algorithm is the possibility to control its performance by changing the key parameters—mainly the cooling rate. The HJ algorithm due to its deterministic nature is much less controllable.

In the case of detecting more than one subgroup of stable points, it is necessary to choose one of them for further calculation of displacements. Deciding which group to select should be preceded by a separate analysis based on a broader range of information, not only of geometrical character.

## Figures and Tables

**Table 1:** Coordinates of points of the test object 1 for both epochs.

Nr	Epoch 1		Epoch 2	
	X [m]	Y [m]	X [m]	Y [m]
1	1600.0000	130.0000	1599.9841	129.9902
2	1200.0000	270.0000	1199.9846	269.9924
3	1360.0000	625.0000	1359.9609	624.9915
4	820.0000	570.0000	819.9836	569.9919
5	823.0000	1100.0000	822.9865	1099.9931
6	1150.0000	2140.0000	1149.9862	2140.0074
7	1520.0000	2175.0000	1519.9856	2175.0127
8	1750.0000	2820.0000	1749.9768	2820.0143
9	1170.0000	2820.0000	1169.9799	2820.0024
10	460.0000	2890.0000	459.9760	2889.9932

**Table 2:** The identification of the stability of reference system by congruency test for the test object 1.

Iteration no.	Group of Points	σ_xy_	σ_xy max_	Point Rejected
1	1 2 3 4 5 6 7 8 9 10	4.41	1.27	3
2	1 2 4 5 6 7 8 9 10	3.47	1.29	7
3	1 2 4 5 6 8 9 10	3.23	1.31	8
4	1 2 4 5 6 9 10	2.60	1.34	6
5	1 2 4 5 9 10	1.84	1.37	9
6	1 2 4 5 10	1.20	1.42	-

**Table 3:** Results of identification of the stability of reference system by the simulated annealing method for the test object 1.

Group of Points	Number of Hits	% Hits	σ_o_ [mm]
1 2 4 5 10	446	44.6	2.39
6 7 8 9 10	554	55.4	2.12

**Table 4:** Results of identification of the stability of reference system by the Hooke-Jeeves method for the test object 1.

Group of Points	Number of Hits	% Hits	σ_o_ [mm]
1 2 4 5 10	623	62.3	2.39
6 7 8 9 10	367	36.5	2.12
3 10	4	0.4	2.83
5 9 10	3	0.3	3.45
3 9	2	0.2	6.52
2 4 5 9 10	1	0.1	3.51

**Table 5:** Coordinates of points of the test object 2 for both epochs.

Nr	Epoch 1		Epoch 2	
	X [m]	Y [m]	X [m]	Y [m]
1	832.011	1627.014	831.940	1626.952
2	682.656	1701.691	682.588	1701.638
3	890.686	1771.035	890.629	1770.962
4	687.990	1829.710	687.939	1829.665
5	821.343	1893.719	821.309	1893.653
6	922.691	1952.394	922.598	1952.398
7	746.665	1995.067	746.568	1995.095
8	890.686	2112.417	890.605	2112.437
9	778.670	2149.756	778.585	2149.781
10	832.011	2229.767	831.957	2229.776
11	1002.702	2149.756	1002.754	2149.755
12	997.368	2251.104	997.410	2251.057
13	1082.714	2288.442	1082.662	2288.483
14	928.025	2331.115	927.973	2331.149
15	1040.041	2379.122	1039.982	2379.156
16	992.034	2491.138	991.975	2491.168
17	1184.062	2491.138	1184.121	2491.136

**Table 6:** Results of identification of the stability of reference system by the simulated annealing method—test object 2.

Group of Points	Number of Hits	% Hits	σ_o_ [mm]
13 14 15 16	751	75.1	3.26
6 7 8 9	208	20.8	5.48
1 2 3 4 5	41	4.1	4.28

**Table 7:** Results of identification of the stability of reference system by the Hooke-Jeeves method for the test object 2.

Group of Points	Number of Hits	% Hits	σ_o_ [mm]
13 14 15 16	589	58.9	3.26
14 15 16	95	9.5	3.29
7 8 9	90	9.0	3.62
6 8 9	45	4.5	6.61
8 9	32	3.2	3.80
6 7 8 9	30	3.0	5.48
1 2 3 4	18	1.8	3.50
10 13 15 16	15	1.5	6.35
1 2	15	1.5	0.95
1 2 3 4 5	14	1.4	4.28
6 10	13	1.3	5.21
15 16	10	1.0	2.60
2 4	9	0.9	6.15
6 7 10	6	0.6	5.71
1 2 4	3	0.3	4.09
13 15 16	3	0.3	3.57
10 13 15	3	0.3	5.12
8 16	2	0.2	10.85
1 2 3	2	0.2	3.19
4	2	0.2	-
7 9 10	1	0.1	8.08
10 15 16	1	0.1	7.17
1 3 4 5	1	0.1	3.59
6	1	0.1	-

## Data Availability

The detailed reports can be accessed upon request from the author.

## References

[1] Amiri-Simkooei A.R. (2001). Strategy for designing geodetic network with high reliability and geometrical strength. J. Surv. Eng..

[2] Nowak E., Odziemczyk W. (2018). Control network reliability reconstruction for Zatonie dam. Rep. Geod. Geoinf..

[3] Zaczek-Peplinska J., Kowalska M.E., Malowany K., Malesa M. (2015). Application of digital image correlation and geodetic displacement measuring methods to monitor water dam behavior under dynamic load. J. Civil Eng. Archit..

[4] Fraser C., Gruendig L. The Analysis of Photogrammetric Deformation Measurements on Turtle Mountain. http://www.asprs.org/wp-content/uploads/pers/1985journal/feb/1985_feb_207-216.pdf.

[5] Zheng W.J., Zhang P.Z., He W.G., Yuan D.Y., Shao Y.X., Zheng D.W., Ge W.P., Min W. (2013). Transformation of displacement between strike-slip and crustal shortening in the northern margin of the Tibetan Plateau: Evidence from decadal GPS measurements and late Quaternary slip rates on faults. Tectonophysics.

[6] Setan H., Singh R. (2001). Deformation analysis of a geodetic monitoring network. Geomatica.

[7] Setan H., Som Z.A.M., Idris K.M. Deformation detection of lightweight concrete block using geodetic and non-geodetic methods. Proceedings of the 11th FIG Symposium on Deformation Measurements.

[8] Bryś H., Przewłocki S. (1998). Geodezyjne Metody Pomiarów Przemieszczeń Budowli.

[9] Teskey W.F., Porter T.R., Chrzanowski A., Wells W. (1988). An integrated method for monitoring the deformation behavior of engineering structures. Proceedings of the 5th International (FIG) Symposium on Deformation Measurements and 5th Canadian Symposium on Mining Surveying and Rock Deformation Measurements.

[10] Chrzanowski A. Geotechnical and other non-geodetic methods in deformation measurements. Proceedings of the Deformation Measurements Workshop.

[11] Muszyński Z., Rybak J., Kaczor P. (2018). Accuracy Assessment of Semi-Automatic Measuring Techniques Applied to Displacement Control in Self-Balanced Pile Capacity Testing Appliance. Sensors.

[12] Chrzanowski A., Chen Y.Q., Secord J.M. Geometrical analysis of deformation surveys. Proceedings of the Deformation Measurements Workshop.

[13] Prószyński W., Kwaśniak M. (2015). Podstawy Geodezyjnego Wyznaczania Przemieszczeń: Pojęcia i Elementy Metodyki.

[14] Chrzanowski A., Wilkins R. Accuracy evaluation of geodetic monitoring of deformations in large open pit mines. Proceedings of the 12th FIG Symposium on Deformation Measurements.

[15] Amiri-Simkooei A.R., Alaei-Tabatabaei S.M., Zangeneh-Nejad F., Voosoghi B. (2016). Stability analysis of deformation-monitoring network points using simultaneous observation adjustment of two epochs. J. Surv. Eng..

[16] Nowel K., Kamiński W. (2014). Robust estimation of deformation from observation differences for free control networks. J. Geod..

[17] Duchnowski R., Wiśniewski Z. (2014). Comparison of two unconventional methods of estimation applied to determine network point displacement. Surv. Rev..

[18] Adamczewski Z. (1979). Algorytm numerycznej kontroli przylegania obiektów. Geodezja i Kartografia.

[19] Niemeier W. (1979). Zur Kongruenz Mehrfach Beobachteter Geodätischer Netze.

[20] Van Mierlo J. (1978). A testing procedure for analysing geodetic deformation measurements. Proceedings of the 2nd International Symposium on Deformation Measurements by Geodetic Methods, Bonn, Germany.

[21] Denli H.H., Deniz R. (2003). Global congruency test methods for GPS networks. J. Surv. Eng..

[22] Denli H.H. (2008). Stable point research on deformation networks. Surv. Rev..

[23] Chen Y.Q. Analysis of Deformation Surveys–A Generalized Method. http://www2.unb.ca/gge/Pubs/TR94.pdf.

[24] Mrówczyńska M. (2010). Identyfikacja układu odniesienia sieci niwelacyjnej obszaru Legnicko-Głogowskiego Okręgu Miedziowego. Acta Geod. Descr. Terr..

[25] Prószyński W. (2010). Problem of partitioned bases in monitoring vertical displacements for elongated structures. Geod. Cartogr..

[26] Wujanz D., Avian M., Krueger D., Neitzel F. (2018). Identification of stable areas in unreferenced laser scans for automated geomorphometric monitoring. Earth Surf. Dynam..

[27] Neitzel F. (2004). Identifizierung Konsistenter Datengruppen am Beispiel der Kongruenzuntersuchung Geodätischer Netze.

[28] Neitzel F. (2005). Die Methode der maximalen Untergruppe (MSS) und ihre Anwendung in der Kongruenzuntersuchung geodätischer Netze. ZfV.

[29] Lehmann R., Lösler M. (2017). Congruence analysis of geodetic networks–hypothesis tests versus model selection by information criteria. J. Appl. Geod..

[30] Hough P.V. Method and Means for Recognizing Complex Patterns. https://patents.google.com/patent/US3069654/en.

[31] Metropolis N., Rosenbluth A.W., Rosenbluth M.N., Teller A.H., Teller E. (1953). Equation of state calculations by fast computing machines. J. Chem. Phys..

[32] Kirkpatrick S., Gelatt C.D., Vecchi M.P. (1983). Optimization by simulated annealing. Science.

[33] Van Laarhoven P.J.M., Aarts E.H.L. (1987). Simulated Annealing: Theory and Applications.

[34] Odziemczyk W. (2020). Application of Simulated Annealing Algorithm for 3D Coordinate Transformation Problem Solution. Open Geosci..

[35] Aarts E.H., Korst J.H., van Laarhoven P.J. (1988). A quantitative analysis of the simulated annealing algorithm: A case study for the traveling salesman problem. J. Stat. Phys..

[36] Malek M., Guruswamy M., Pandya M., Owens H. (1989). Serial and parallel simulated annealing and tabu search algorithms for the traveling salesman problem. Ann. Oper. Res..

[37] Yetkin M. (2013). Metaheuristic optimisation approach for designing reliable and robust geodetic networks. Surv. Rev..

[38] Yetkin M., Berber M. (2014). Implementation of robust estimation in GPS networks using the artificial bee colony algorithm. Earth Sci. Inform..

[39] Yetkin M. (2018). Application of robust estimation in geodesy using the harmony search algorithm. J. Spat. Sci..

[40] Berné J.L., Baselga S. (2004). First-order design of geodetic networks using the simulated annealing method. J. Geod..

[41] Baselga S. (2011). Second order design of geodetic networks by the simulated annealing method. J. Surv. Eng..

[42] Baselga S., García-Asenjo L. (2008). Global robust estimation and its application to GPS positioning. Comput. Math. Appl..

[43] Baselga S., Klein I., Suraci S.S., de Oliveira L.C., Matsuoka M.T., Rofatto V.F. (2020). Performance comparison of least squares, iterative and global L1 norm minimization and exhaustive search methods for outlier detection in leveling networks. Acta Geodyn. Geomater..

[44] Jia F., Lichti D. (2017). A Comparison of Simulated Annealing, Genetic Algorithm and Particle Swarm Optimization in Optimal First-Order Design of Indoor TLS Networks. ISPRS Ann. Photogramm. Remote Sens. Spat. Inf. Sci..

[45] Hooke R., Jeeves T.A. (1961). "Direct Search" Solution of Numerical and Statistical Problems. J. ACM.

[46] Mazouz L., Zidi S.A., Hafaifa A., Hadjeri S., Benaissa T. (2019). Optimal Regulators Conception for Wind Turbine PMSG Generator Using Hooke Jeeves Method. Period. Polytech. Elec. Eng. Comp. Sci..

[47] Torres R.H., de Campos Velho H.F., Chiwiacowsky L.D. (2019). Rotation-Based Multi-Particle Collision Algorithm with Hooke–Jeeves Approach Applied to the Structural Damage Identification. Computational Intelligence, Optimization and Inverse Problems with Applications in Engineering.

[48] Shakya A., Mishra M., Maity D., Santarsiero G. (2019). Structural health monitoring based on the hybrid ant colony algorithm by using Hooke–Jeeves pattern search. SN Appl. Sci..

[49] Nagina F., Saeed M., Tabassum M.F., Ali J. (2016). Solution of quarter car model by pattern search methods. Sci. Int..

[50] Kruczkowski M. (2019). Identification of theoretical parameters used to forecast impact of underground mining on one coal seam performed on the basis of a geodetic survey. IOP Conference Series: Earth and Environmental Science.

[51] Zeng H., Yi Q., Wu Y. (2016). Iterative approach of 3D datum transformation with a non-isotropic weight. Acta Geod. Geophys..

